# Environmental Microbiology: Control of Foodborne and Waterborne Diseases Course — January 6–11, 2014

**Published:** 2013-11-01

CDC and Emory University’s Rollins School of Public Health will cosponsor a 6-day course on Environmental Microbiology: Control of Foodborne and Waterborne Diseases at Emory University, Rollins School of Public Health. This course on the surveillance of foodborne and waterborne diseases is designed for public health practitioners and other students interested in the safety of food and water. It provides a broad overview of the major foodborne and waterborne diseases.

This course describes how information from surveillance is used to improve public health policy and practice in ways that contribute to food and water safety. Discussions focus on the microorganisms and chemical agents responsible for food and water-transmitted diseases, the diseases they cause, pathogenesis, clinical manifestations, reservoirs, modes of transmission, and surveillance systems. The transport, survival, and fate of pathogens in the environment, the concept of indicator organisms as surrogates for pathogens, and the removal and inactivation of pathogens and indicators by water and wastewater treatment processes will be analyzed. Examples of the public health impact of quality assurance programs, such as hazard analysis and critical control points, on control foodborne and waterborne diseases in industrialized and developing countries will be highlighted.

This course is offered to matriculating students at Emory University and to nonmatriculating public health professionals. Tuition will be charged. The application deadline is December 15, 2013, or until all slots have been filled. Additional information and applications are available by mail (Emory University, Hubert Department of Global Health [Attn: Pia Valeriano], 1518 Clifton Rd. NE, Rm. CNR Bldg., Room 7038, Atlanta, GA 303220), by telephone (404-727-3485), online (http://www.sph.emory.edu/epicourses), or by e-mail (pvaleri@emory.edu).

